# Efficacy and Safety of Eculizumab in the Treatment of Transplant-Associated Thrombotic Microangiopathy: A Systematic Review and Meta-Analysis

**DOI:** 10.3389/fimmu.2020.564647

**Published:** 2021-01-20

**Authors:** Rui Zhang, Meng Zhou, Jiaqian Qi, Wenjing Miao, Ziyan Zhang, Depei Wu, Yue Han

**Affiliations:** ^1^Jiangsu Institute of Hematology, The First Affiliated Hospital of Soochow University, Suzhou, China; ^2^Collaborative Innovation Center of Hematology, Soochow University, Suzhou, China; ^3^Institute of Blood and Marrow Transplantation, Suzhou, China; ^4^Key Laboratory of Thrombosis and Hemostasis of Ministry of Health, Suzhou, China; ^5^National Clinical Research Center for Hematologic Diseases, Suzhou, China; ^6^State Key Laboratory of Radiation Medicine and Protection, Soochow University, Suzhou, China

**Keywords:** Eculizumab, terminal complement inhibitor, transplant-associated thrombotic microangiopathy, hematopoietic stem cell transplantation, efficacy, safety, meta-analysis

## Abstract

**Background:**

Transplant-associated thrombotic microangiopathy (TA-TMA) is a dangerous and life-threatening complication in patients undergoing hematopoietic stem cell transplantation (HSCT). Eculizumab has been used in the treatment of TA-TMA, and several studies have confirmed the benefit of Eculizumab in patients with TA-TMA. However, the results remain controversial. We conducted a systematic review and meta-analysis to evaluate the efficacy and safety of Eculizumab for TA-TMA.

**Materials and Methods:**

We searched PubMed and Embase for studies on the efficacy and safety of Eculizumab in TA-TMA patients. Efficacy outcomes consisted of overall response rate (ORR), complete response rate (CRR), and survival rate at the last follow-up (SR). Safety outcomes were adverse events (AEs), including infection, sepsis, impaired liver function, infusion reactions, and death.

**Results:**

A total of 116 patients from six studies were subjected to meta-analysis. The pooled estimates of ORR, CRR, and SR for TA-TMA patients were 71% (95% CI: 58–82%), 32% (95% CI: 11–56%), and 52% (95% CI: 40–65%), respectively. Only one patient presented with a severe rash, and infection was the most common AEs. The main causes of death were infection and GvHD.

**Conclusion:**

Current evidence suggests that Eculizumab improves SR and ORR in patients with TA-TMA and that Eculizumab is well tolerated. However, the number of studies is limited, and the findings are based mainly on data from observational studies. Higher quality randomized controlled trials and more extensive prospective cohort studies are needed.

## Introduction

Hematopoietic stem cell transplantation (HSCT) is a recognized treatment for both malignant and non-malignant diseases. While this treatment has increased cure rates and reduced disease mortality, its complications remain life-threatening and of concern. Transplant-associated thrombotic microangiopathy (TA-TMA) is one of the most devastating complications of hematopoietic stem cell transplantation. A recent study reported a 3-year cumulative incidence rate of 3% for TA-TMA, and TA-TMA was associated with high mortality (HR = 3.1, 95% CI: 2.8–16.3%) (1). Treatment intensity, use of calcitonin inhibitors (CNIs), graft-*versus*-host disease (GvHD), and viral infection are risk factors for TA-TMA (2, 3). Patients with TA-TMA are characterized by microangiopathic hemolytic anemia, unexplained thrombocytopenia, elevated lactate dehydrogenase (LDH), and endothelial injury-related organ failure, such as hypertension, chronic kidney disease (CKD), pulmonary hypertension, gastrointestinal or central nervous system disease (4). TA-TMA is mainly defined with two standard diagnostic criteria. One is the International Working Group (IWG) (thrombocytopenia in the blood; new-onset, prolonged or progressive thrombocytopenia; sudden and persistent elevation of LDH; decreased hemoglobin or increased transfusion requirements, decreased serum hemoglobin) (5), and the other is overall-TMA (O-TMA) as demonstrated by Cho et al. (Elevated LDH; new-onset thrombocytopenia with a platelet count <50 × 10^9^/L or thrombocytopenia >50%; new-onset anemia with hemoglobin below the lower limit of normal or requiring transfusion support; the presence of typed cells in peripheral blood or histological evidence of microangiopathy in tissue specimens; no coagulation dysfunction, negative Coombs test) (6). The mechanism of how systemic microvascular endothelial injury leads to TA-TMA remains unclear. Due to its similar histomorphology to atypical hemolytic uremic syndrome (aHUS), most studies suggest that it is dysregulation of the complement system that causes TA-TMA to occur (7–9). Subsequently, C5 is cleaved to C5a and C5b, forming a cell membrane attack complex (MAC, C5b-9) on the surface of the endothelial cells, resulting in further endothelial cell damage (8). Significantly elevated plasma C3b, sC5b-9 levels were observed in TA-TMA patients (10).

There is no consensus on care strategies for TA-TMA. Conventional treatments, including supportive care measures, withdrawal of CNIs, therapeutic plasma exchange (TPE) and pharmacological treatments such as rituximab, defibrotide, and daclizumab have been used in the treatment of TA-TMA (8). Recently, the use of Eculizumab for the treatment of TA-TMA has raised concerns. With more and more case reports reaching remission (11–13), Eculizumab has shown its benefits in the treatment of patients with TA-TMA. Eculizumab is a terminal complement inhibitor that works by inhibiting the cleavage of C5 to C5a and C5b. C5b-9 is then blocked from forming on the surface of endothelial cells (14). Since the FDA approved Eculizumab for the treatment of atypical hemolytic uremic syndrome (aHUS) (15), TA-TMA patients treated with Eculizumab were treated according to the standard regimen of aHUS (16). Patients received induction therapy with Eculizumab 900mg once a week for four weeks. When hematological signs of TA-TMA resolved, maintenance therapy was continued with 1,200 mg given every two weeks (17, 18). Some researchers have paid attention to evaluate the therapeutic benefits of Eculizumab in patients with TA-TMA. A study by Joslyn et al. showed a hematological response rate of 70% in patients with TA-TMA after Eculizumab treatment (19), similar to the 67% reported by Michelle et al. (20), but much worse compared to the 93% reported by Stephan et al. (21). In addition, the survival rate of 60% (19) studied by Joslyn et al. was similar to that of Michelle et al.’s 67% (20) and more favorable than that of Stephan et al. (33%) (21). The efficacy results vary from study to study.

To determine which factors may contribute to diversity in response rates and survival, we systematically reviewed relevant studies of Eculizumab in patients with TA-TMA and performed a meta-analysis to better understand the efficacy and safety of Eculizumab.

## Materials and Methods

### Search Strategy

PubMed and Embase databases were searched from their inception up to February 15, 2020, for relevant studies, and publication language was restricted as English. The search strategy was based on the following combined MeSH terms: ((((“Transplantation”[Mesh]) OR Transplantations[Title/Abstract])) AND ((“Thrombotic Microangiopathies”[Mesh]) OR (((Microangiopathies, Thrombotic[Title/Abstract]) OR Microangiopathy, Thrombotic[Title/Abstract]) OR Thrombotic Microangiopathy[Title/Abstract]))) AND ((((((((Eculizumab) OR Alexion) OR Soliris) OR 5G1.1) OR H5G1.1VHC+H5G1.1VLC) OR H5G1.1) OR H5G1-1) OR H5G11). The systematic review and meta-analysis were conducted and reported in compliance with the PRISMA statement (22).

### Selection Criteria

Studies eligible in the meta-analysis met the following criteria: (1) Patients developed TMA after hematopoietic stem cell transplantation; (2) Eculizumab was regarded as first-line therapy or second-line therapy; (3) studies are cohort studies and data from case, letter, review, conference abstract were not taken into consideration. (4) Outcomes of this meta-analysis will include complete response, overall response, survival rate, and adverse events (AEs). To minimize bias in the selected pieces of literature, each paper with a title and a general meeting, our inclusion criteria were checked by two reviewers independently. Then full texts were identified and reevaluated carefully. Any disagreements were further discussed and resolved by consulting a senior investigator to reach a consensus.

### Outcome Measures

The diagnosis of TA-TMA was identified according to IWG (5) or O-TMA (6). Hematological response (HR) was defined as disappearance of schistocytes, normalization of LDH and haptoglobin, and dependence of transfusion. Complete response (CR) was defined as hematological response with resolution of organ dysregulation caused by TMA. Among outcomes of patients undergoing Eculizumab therapy, which included hematological response (HR), complete response (CR) and no response (NR), an effective overall response (OR) was composed of CR and HR. The survival rate (SR) was evaluated at the last follow-up of each study. Adverse events were reported at baseline and a follow-up visit with a focus on meningococcal infections, serious infections, sepsis, hepatic impairment, infusion reaction, and death.

### Data Extraction

We extracted general characteristics, including the surname of the first author, year of publication, setting, sample size from each included study. Pretreatment patient data collected included age, gender, primary disease, type of transplant, diagnostic criteria, the level of serum sC5b-9, time from HSCT to TA-TMA diagnosis, time from TA-TMA diagnosis to Eculizumab use. Treatment variables included median days of Eculizumab therapy, median Eculizumab dose, outcomes and prognosis.

### Quality Assessment

The Newcastle–Ottawa Quality Assessment Scale for Cohort Studies was applied to evaluate the quality and risk of bias of included studies (23).

### Statistical Analysis

Efficacy was evaluated by the overall response rate (ORR), complete response rate (CRR), survival rate (SR). Safety of Eculizumab was evaluated by adverse events (AEs) including treatment-emergent adverse events (TEAEs), treatment-related adverse events (TRAEs), serious adverse events (SAEs), and cause of death. All the raw data extracted from the studies were transformed with the Freeman–Tukey double arcsine method. Estimated proportions (ES) with 95% confidence intervals (CIs) were calculated for ratio outcomes. The presence of heterogeneity was assessed by using the chi-square test of heterogeneity and the I^2^ measure of inconsistency. Higher I^2^ value and lower P-value indicate a greater degree of heterogeneity, and I^2^ values ≤25%, between 25 and 50%, and ≥50% were equal to low, median, and substantial heterogeneity, respectively. A random-effects model was used regardless of heterogeneity. Considering some significant factors might affect clinical response, survival and prognosis, subgroup analyses and meta-regression for the overall response rate (ORR) and survival rate (SR) were performed based on publication year, setting, sample size, age, primary disease, median days between transplant to TA-TMA, Eculizumab as first-line therapy, overall median therapy duration, the median number of Eculizumab doses if relevant data were available. The p-value of meta-regression of publication <0.05 accounted for the existence of heterogeneity. Funnel plots were inappropriate to perform as the total number of included studies were six (<10). Sensitivity analyses were conducted further to decide the stability and reliability of the results we performed by deletion of every single investigation. All statistical analyses were conducted using R (version 3.6.2). A two-tailed P value of less than 0.05 was considered statistically significant.

## Results

### Data Sources

**Figure 1:**
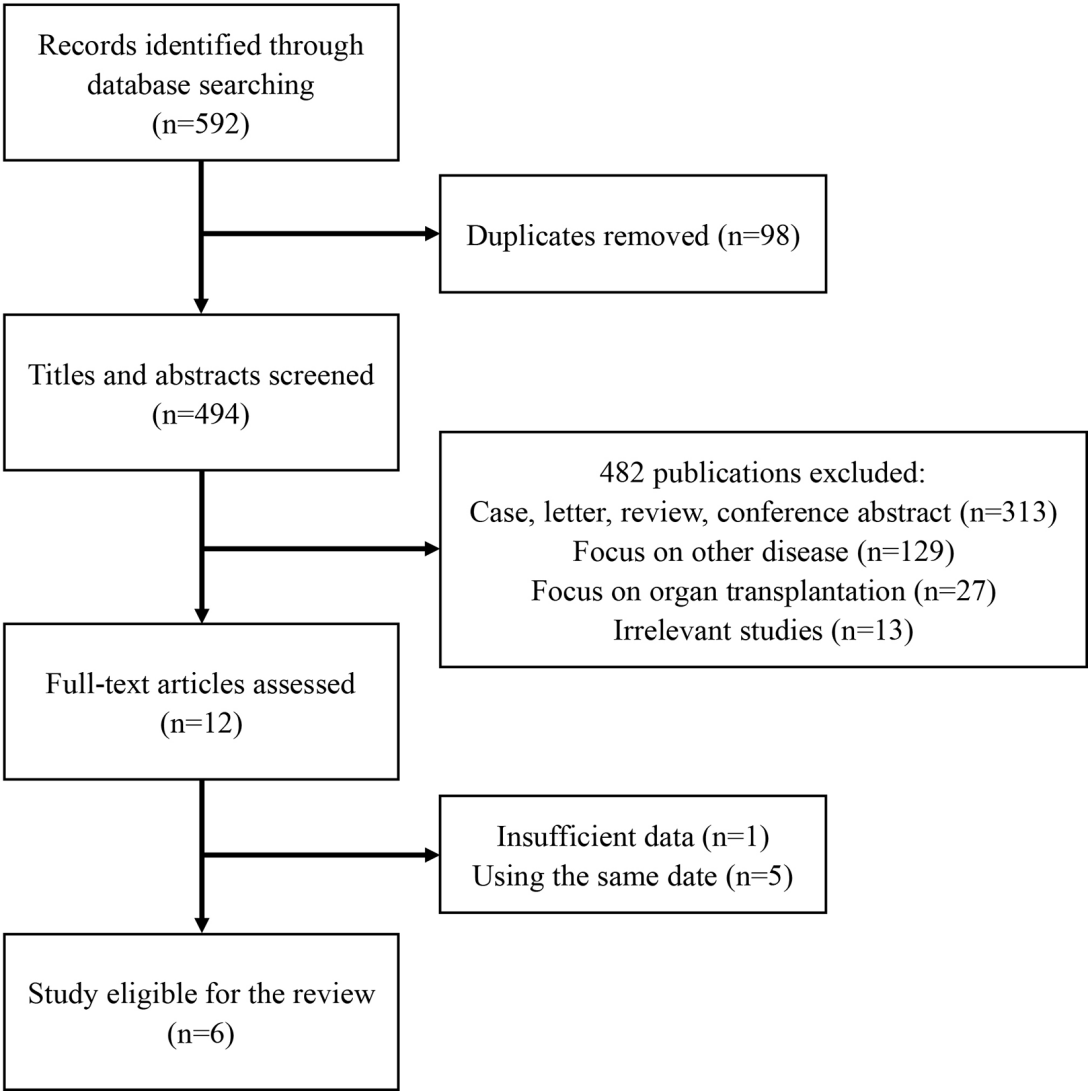
Preferred Reporting Items for Systematic Reviews and Meta-Analyses analysis.

In all, 592 publications were initially identified based on literature search parameters (**Figure 1**). A total of 98 were discarded for duplicates, and 482 records were removed by inspecting the titles and abstracts based on prospective search criteria. After full-text evaluations of the remaining 12 articles, six were considered to be eligible for the systematic review and meta-analysis according to the selection criteria (19–21, 24–26). The necessary information of the included six articles was summarized in **Table 1**. These articles were published, ranging from 2015 to 2020. TA-TMA was diagnosed mainly by adopting O-TMA proposed by Cho et al. Treatment with Eculizumab was primarily administered following the recommended dose for aHUS. And for pediatric patients, the dose of Eculizumab followed the protocol of Jodele et al. Baseline characteristics of patients including age, gender, primary disease, type of transplant and Eculizumab treatment, and outcomes of efficacy and safety endpoints were described in **Table 2**. TA-TMA was diagnosed at a median age of 23 years (range1.2–66) post-transplantation. Most patients had transplant performed for hematological malignancy, neuroblastoma, as well as immune deficiency. Of patients with available information, 82.8% received allogeneic hematopoietic cell transplantation, 69.9% received CNI treatment at the time of diagnosis, aGvHD occurred in 39.7%, and viral infection was 26.7%.

**Table 1:** Study from which patient-level data were provided and included in meta-analysis.

Study	Year	setting	diagnostic criteria	Patients with TMA after HSCT in the study	TA-TMA patients with Eculizumab treatment in the study	Eculizumab regime
Flore et al. (24)	2015	Multicenter	O-TMA criteria	12	12	Induction therapy: 900 mg weekly for 4 weeks Maintenance therapy: 1,200 mg every 2 weeks
Prajwal et al. (25)	2016	Multicenter	O-TMA criteria	9	9	Adult patients: induction therapy was 900 mg weekly for 4 weeks, followed by 1,200 mg every 2 weeks for maintenance therapy. Pediatric patients: the first dose was based on weight, subsequent dose according to CH50.
Stephan et al. (21)	2017	Single-center	O-TMA criteria	39	15	Induction therapy: 900 mg weekly for 4 weeks Maintenance therapy: 1,200 mg every 2 weeks
Joslyn et al. (19)	2018	Single-center	O-TMA criteria	10	10	Induction therapy: 900 mg weekly for 4 weeks Maintenance therapy: 1,200 mg every 2 weeks
Michelle et al. (20)	2019	Single-center	Before 2010, IWG criteria After 2010, O-TMA criteria	9	6	Adult patients: induction therapy was 900 mg weekly for 4 weeks, followed by 1,200 mg every 2 weeks for maintenance therapy. Pediatric patients: the first dose was based on weight, subsequent dose according to CH50.
Sonata et al. (26)	2020	Single-center	O-TMA criteria	177	64	The first dose was based on weight, subsequent dose according to CH50.

CH50, a total hemolytic complement activity level; HSCT, Hematopoietic stem cell transplantation; IWG, International Working Group; O-TMA, overall-TMA; TA-TMA, Transplant associated thrombotic microangiopathy.

**Table 2:** Baseline characteristics for patients included in meta-analysis.

Variable	Flore et al. (n = 12)	Prajwal et al. (n = 9)	Stephan et al. (n = 15)	Joslyn et al. (n = 10)	Michelle et al.(n = 6)	Sonata et al. (n = 64)
Age, median (range)	39(1.2–66)	7(2–61)	48(23–66)	44(17–59)	5.2(2.5–25)	5.5(2.7–11.7)
Gender, male (%)	7(58)	6(67)	7(47)	4(40)	4(67)	40(63)
Primary disease	Hematological disease	Hematological disease/others	Hematological disease	Hematological disease	Hematological disease/others	Hematological disease/others
Type of transplant	Allo/UCB	Allo/Auto	Allo	Allo/UCB	Auto	Allo/Auto/UCB
Conditioning regimen	MAC/RIC	MAC/RIC	MAC/RIC	MAC/RIC	MAC	MAC/RIC
Other risk factors at diagnosis, number (%)						
CNI used	8(67)	7(78)	9(60)	10(100)	NA	49(77)
aGvHD	8(67)	5(56)	12(80)	7(70)	0(0)	14(22)
Affection	6(50)	2(22)	8(67)	8(80)	1(17)	6(9)
Interval between HSCT and diagnosis, median days	121	68	264	93	35	<100**^a^**
sC5b-9	NA	NA	456(127-810)	NA	151.5(100-460)	398(282-544)
Interval between diagnosis and Eculizumab therapy, median days	31	24	10	4	18	NA
Eculizumab therapy, median days	65	178	52.5	48.5	110	66
First-line therapy, number (%)/second-line therapy, number	5(42)/7	2(22)/7	11(73)/4	7(70)/3	6(100)/0	64(100)/0
Eculizumab dose, median dose	6	8	9	6	9.5	11
Overall response, number (%)	6(50)	7(78)	13(93)**^b^**	7(70)	4(67)	41(64)
Complete response, number (%)	2(17)	5(56)	NA	1(10)	1(17)	36(56)
Survivals, number (%)	4(33)	7(78)	5(33)	6(60)	4(67)	35(55)
Median follow-up months	14	12	8	13	30	15
AEs during Eculizumab therapy	Infection	No	Infection	Skin rash	NA	Infection
Cause of death, numbers (%)						
TA-TMA related	4(50)	0	2(20)	0	1(50)	8(28)
Infection	2(25)	0	8(80)	2(50)	0	6(21)
GvHD	2(25)	2(100)	0	1(25)	0	14(48)
Relapse of the primary disease	0	0	0	1(25)	1(50)	1(3)
Prognosis	CKD	CKD	CKD	CKD	CKD/HTN	CKD/HTN

Allo,; Auto, Autologous HSCT; CNI, calcineurin inhibitors; HTN hypertension; MAC, myeloablative regimen; RIC, reduced intensity regimen; UCB, umbilical cord blood.

a As 92% patients were diagnosed TA-TMA at a median of 23 days (IQR 3–48), and five had TA-TMA between 118 and 221 days after transplant, we regarded that the median days of interval between HSCT and diagnosis was less than 100 days.

b one unknown response due to early death.

### Efficacy Outcomes

**Figure 2:**
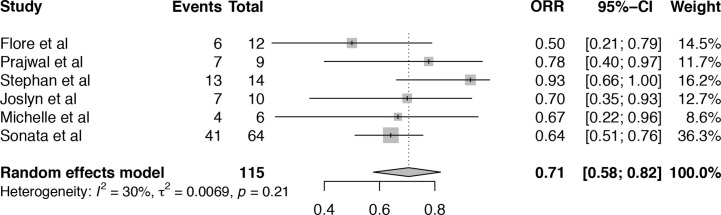
Forest plot of the estimated proportions (95% CI) for overall response rate (ORR) of the TA-TMA patients after Eculizumab treatment.

A total of six articles, including 116 patients were eligible for the analysis of overall response rate (ORR) (19–21, 24–26). It showed that the heterogeneity among the included studies was median (I^2^ = 30%, P = 0.21). Pooled result of ORR in TA-TMA patients treated with Eculizumab was 71% (95%CI: 58–82%) (**Figure 2**). Subgroup analysis and meta-regression were conducted to evaluate the potential effects of setting, sample size, age, primary disease, median days between transplant to TA-TMA, Eculizumab as first-line or second-line, therapy duration, the median number of Eculizumab doses (**Table S1**). Subgroup analysis of setting showed that the pooled ORR of single-center (ORR = 74%, 95%CI:57–88%) was numerically higher than that of multicenter (ORR = 63%, 95%CI:35–87%). Subgroup analysis of the number of Eculizumab dose showed that the pooled ORR of dose ≥8 was 75% (95%CI:58–89%), which is higher than that <8 (ORR = 59%, 95%CI:37–80%). The therapy duration <60 days achieved a higher survival rate (ORR = 84%, 95%CI:57–100%) than that ≥60 days (ORR = 64%, 95%CI:54–75%). However, the p-value of meta-regression of variables were all >0.05, which did not account for the existence of heterogeneity. The application of sensitivity analysis showed that the study by Stephan et al. (21) impacted the overall results (**Figure S1**), which was a historically controlled, single-center study as opposed to the other observational studies.

**Figure 3:**
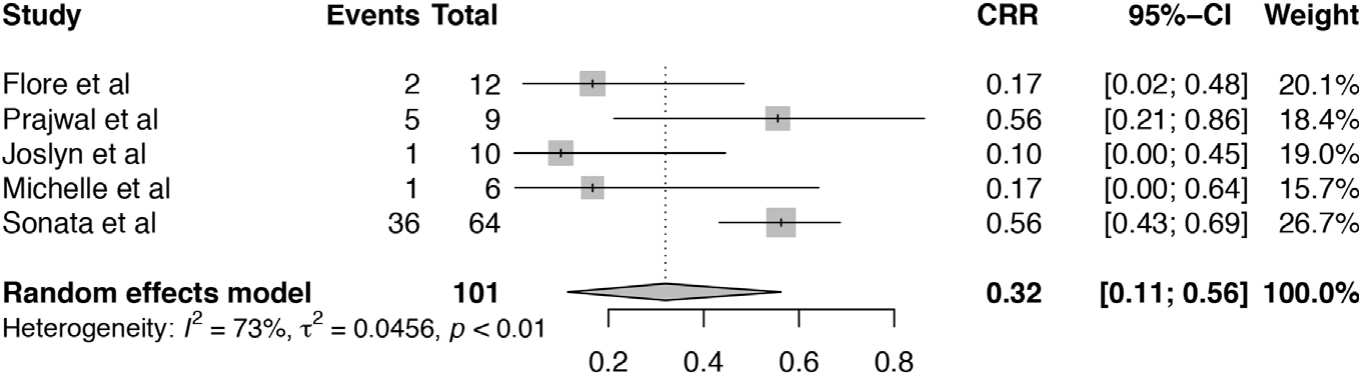
Forest plot of the estimated proportions (95% CI) for complete response rate (CRR) of the TA-TMA patients after Eculizumab treatment.

Among these included studies, five studies reported the complete response rate (CRR) for TA-TMA patients receiving Eculizumab treatment (19, 20, 24–26). The heterogeneity among included studies was substantial (I^2^ = 73%, P < 0.01) and the pooled estimate of CRR was 32% (95%CI: 11–56%), which was much lower than overall response (**Figure 3**). As the number of studies was limited, the source of heterogeneity could not be analyzed by meta-regression. Sensitivity analysis of CRR in TA-TMA patients treated with Eculizumab informed that Sonata et al. (26) might be the source of heterogeneity (**Figure S2**). The CRR of the study was 88% which was much higher than other studies, and it is an observational study consisting of 64 pediatric patients diagnosed as high-risk TA-TMA. All the patients were offered Eculizumab as first-line therapy, and the median number of Eculizumab doses given was 11, which is more than that of other studies.

**Figure 4:**
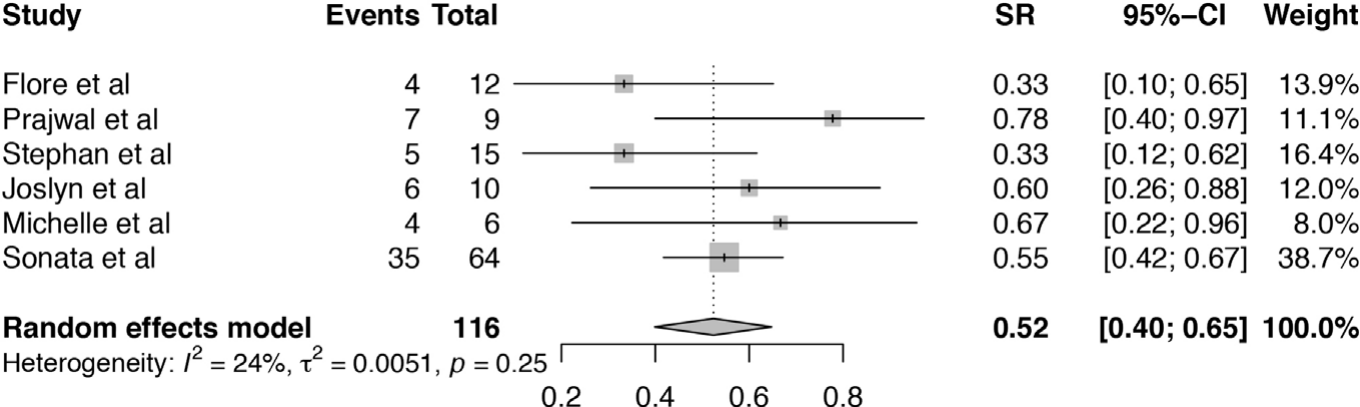
Forest plot of the estimated proportions (95% CI) for survival rate (SR) of the TA-TMA patients after Eculizumab treatment.

The survival rate (SR) of TA-TMA patients treated with Eculizumab was analyzed in six articles (19–21, 24–26). The heterogeneity among the six included studies was low (I^2^ = 24%, P = 0.25). Pooled estimate of SR was 52% (95%CI: 40–65%) (**Figure 4**). Subgroup analysis and meta-regression were conducted to evaluate the potential effects of publication year, setting, sample size, age, primary disease, median days between transplant to TA-TMA, therapy duration, the median number of Eculizumab doses (**Table S2**). Subgroup analysis of sample size showed that the pooled SR of the size ≤10 (SR = 68%, 95%CI:47–86%) was numerically higher than that >10 (SR = 45%, 95%CI:29–61%). Subgroup analysis of age showed that the pooled SR of pediatric TA-TMA patients (SR = 59%, 95%CI:47–70%) was numerically higher than that of adult patients (SR = 40%, 95%CI:24–57%). Subgroup analysis of primary disease indicated that the pooled SR of hematological disease was 40% (95%CI:24–57%), which is lower than that of primary disease containing the hematological disease and others (SR = 59%, 95%CI:47–70%). The pooled SR of TA-TMA diagnosed during first 100 days after transplant was 59% (95%CI:48–70%), which was significantly higher than that of TA-TMA diagnosed more than 100 days after transplant (SR = 33%, 95%CI:16–53%). The therapy duration of more than 65 days achieved a higher survival rate (SR = 59%, 95%CI:47–70%) than that less than 65 days (SR = 40%, 95%CI:24–57%). P-value of median days between transplant and TA-TMA was 0.0266, which can explain the source of heterogeneity. The result of sensitivity analysis indicated that omitting the study of Prajwal et al. (25) and Stephan et al. (21) may influence the pooled results (**Figure S3**).

### Safety Outcomes

All six studies were reported that treatment of TA-TMA with Eculizumab was well tolerated (19–21, 24–26). Among 116 patients, only one case was reported to get severe skin rash leading to drug discontinuation during Eculizumab therapy (19). Three studies were reported that some patients developed the infection after starting Eculizumab therapy for TA-TMA (21, 24, 26), and no meningococcal infections were reported. After the Eculizumab therapy, many survivors suffered from CKD and HTN, and much of them were still depend on dialysis (**Table 2**). As the data from the studies were limited, further studies need to analyze the prognosis.

**Figure 5:**
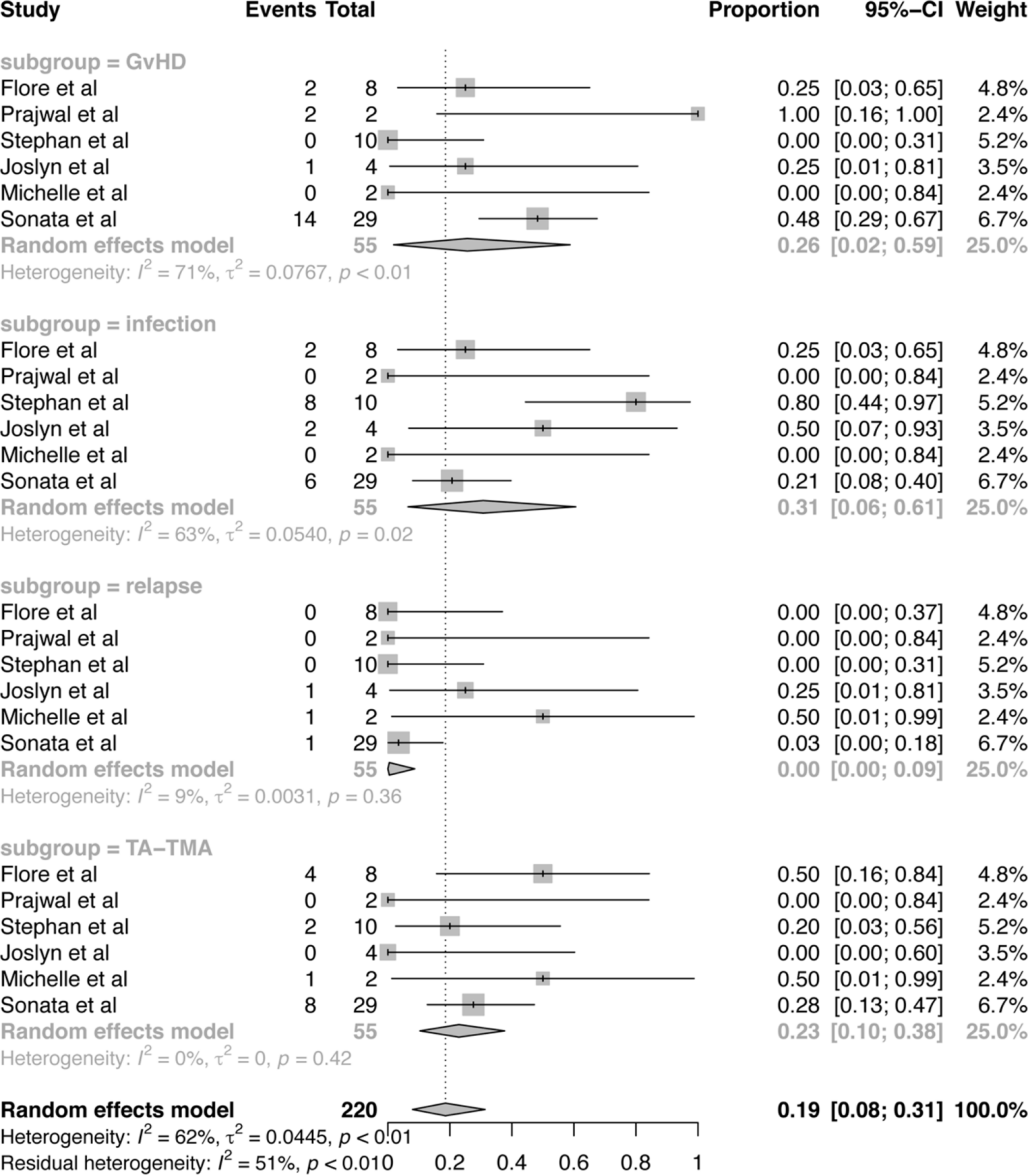
Forest plot of the estimated proportions (95% CI) for cause of death after Eculizumab treatment.

From a total of 55 subjects who died, cause of death can be divided into four risk factors: GvHD, infection, TA-TMA related organ failure, relapse of disease, which were presented in **Figure 5**. Among these risk factors, the proportion of infection was 31% (95%CI:6–61%), which is much higher than the other three factors. And the proportion of GvHD was 26% (95%CI:2–59%). TA-TMA related death was occupied 23% (95%CI:10–38%). Death related to relapse of primary disease was the least. No study was reported that death is related to the use of Eculizumab.

### Risk of Bias

The Newcastle-Ottawa scale was used to assess the risk of bias. The NOS scores of every study ranged from 7 to 9, with an average of 7.7. The detailed information of NOS scores is shown in **Table S3**.

## Discussion

In all six observational studies, including 116 patients, were included in a systematic review and meta-analysis to investigate the efficacy and safety of Eculizumab in patients with TA-TMA (19–21, 24–26). After Eculizumab treatment, almost 71% of patients responded to the therapy. However, the number of patients who reached full response was significantly lower (32%). Patients with TA-TMA treated with Eculizumab had a 52% survival rate at a median follow-up of 13.5 months after HSCT. Previous treatment strategies for TA-TMA after HSCT have focused on conventional therapies, including withdrawal of CNIs, plasmapheresis, defibrillation, rituximab, and combinations of several therapies (8). A retrospective study showed that 24% of TA-TMA patients (n = 33) underwent plasmapheresis and achieved a clinical response with an SR of 45% at 100 days after diagnosis (27). Corti et al. reported a total of 12 TA-TMA patients undergoing defibrillation in two centers with an ORR and SR of 67 and 50%, respectively (28). Another study by Au et al. showed an ORR of 80% (n = 4/5) for rituximab treatment and a study SR of 60% at a median follow-up of 305 (250–440) d (29). Due to the small sample size reported above, it is difficult to perform a systematic and comprehensive comparison between conventional therapy and Eculizumab. Nonetheless, the summary ORR and SR of Eculizumab from our meta-analysis appeared higher than traditional treatment.

A retrospective study by Prajwal et al. provided an evaluation of efficacy for TA-TMA patients treated with Eculizumab (25). Although they also demonstrated higher response rates and survival in TA-TMA patients treated with Eculizumab, the articles they included were mostly cases (11–13). Cases usually report successful treatment rather than unsuccessful treatment. The benefit of Eculizumab may be significantly overestimated, and it is difficult to explain the heterogeneity between cases. In addition, this retrospective analysis focused on patients with refractory discontinuation of calcineurin inhibitors and plasma exchange in patients with TA-TMA. Eculizumab was considered second-line therapy, whereas patients receiving Eculizumab as first-line therapy were not considered. What’s more, adverse events during Eculizumab treatment were not analyzed. Our current meta-analysis incorporates the largest number of observational studies to date. Among these included studies, we joined the largest cohort to date, which included the terminal complement blocker Eculizumab as first-line treatment in patients with TA-TMA, which provides a higher weighting in the meta-data. Subgroup analysis and meta-regression were performed to detect heterogeneous sources. A sensitivity analysis was performed to demonstrate the stability and reliability of the findings. More importantly, our study provides not only a pooled assessment of efficacy but also of AEs and cause of death. Therefore, the current meta-analysis is a more comprehensive and credible analysis of the effectiveness and safety of Eculizumab for TA-TMA.

Because efficacy outcomes vary widely across centers, our current study analyzed which factors may contribute to differences in response rates and survival rates. Since the p-values of meta-regression were all greater than 0.05, the subgroup analysis of ORR could not explain the source of the difference. Flore et al. (24) studied the lowest ORR compared to four other studies (19–21, 26). Due to the multicenter and retrospective nature of the study, there was heterogeneity in patient inclusion criteria, primary disease, stem cell transplantation characteristics, and Eculizumab regimens. It seems that this setup may be the source of the ORR discrepancy. More importantly, the overall response in the study results was good, but the complete response rate was meagre. This indicates that the kidney damage is advanced by the time of Eculizumab treatment. As a result, most patients do not get organ recovery. From this, it appears that the delay in Eculizumab treatment may prevent patients from achieving optimal response and maximum recovery of organ function. However, in the study by Joslyn et al., the median duration between diagnosis and Eculizumab therapy was shorter, at four days, compared to other studies. Only one patient achieved a complete response and organ function was restored (19). This suggests that earlier initiation of Eculizumab may have no significant effect on restoring organ function. How to improve the full response rate remains a big question. Our subgroup analysis of TA-TMA patient survival revealed that the time between HSCT and TA-TMA diagnosis is a potential source of SR heterogeneity, as the p value for meta-regression for days between transplantation and TA-TMA is 0.0266. TA-TMA diagnosed within the first 100 days after transplantation is more susceptible to publication bias than TA-TMA more than 100 days after transplantation. Therefore, early diagnosis of TA-TMA is the key to successful treatment of TA-TMA. However, the early diagnosis of TA-TMA faces challenges due to overlapping clinical features and the lack of standard diagnostic criteria as most studies support the idea that unregulated complement activation leads to the development of TA-TMA occurrence (7–9). The search for sensitivity and specificity of complement activation monitoring biomarkers should be of interest. Recently, Orsolya et al. showed that early elevation of sC5b-9 is a predictor of late development of TA-TMA (30). In this study, sC5b-9 levels increased from baseline levels to day 28 in patients with TA-TMA (n = 10), while the same trend was observed in only nine patients (p = 0.031) without TA-TMA (n = 23). In our meta-analysis, sC5b-9 levels were documented in three articles, and elevated sC5b-9 was observed in TA-TMA patients in two studies. However, the timing of the detection of sC5b-9 levels was not elaborated. Further studies are needed to determine whether terminal pathway activation is an independent predictor of TA-TMA after HSCT. Median age and primary disease may be another two factors contributing to significant differences between studies though their p-values of meta-regression are both 0.0827. Children seem to achieve higher SR than adults. In addition, SR of TA-TMA patients under treatment of Eculizumab reported in the studies which focused on hematological disease is lower than that of other studies. Sensitivity analysis of ORR and SR showed that the investigation by Stephan et al. (21) was a source of heterogeneity in SR of this meta-analysis. The SR (33%) in this single-center analysis was significantly lower compared to other studies. Patients in this study were diagnosed as hematological disease and the median age was 48 years, which was older than that of other five reports included. Additionally, the median days from hematopoietic stem cell transplantation to TA-TMA diagnosis were 264 days, the longest in all the studies.

The safety profile seems to indicate that Eculizumab is well tolerated. A more substantial observational data set covering a 5-year registry of patients with aHUS reported that no new safety issues were identified in patients treated with adult or pediatric Eculizumab (31). In our meta-analysis, only one case of TRAEs, *i.e.* one patient with a severe rash, was reported, resulting in discontinuation of Eculizumab therapy. The most commonly reported AEs are infections. Eculizumab is a monoclonal antibody that inhibits C5 cleavage and prevents terminal complement activation (4). Patients treated with Eculizumab have an increased risk of infection, especially meningococcal infections, due to the lack of adequate functional complement (17). Whereas a study by Sonata et al. reported no cases of meningococcal infections in patients who had not received the meningococcal vaccine (32). And in our present study, no meningococcal infections were reported, which corresponds to the findings of Sonata et al. However, among patients treated with Eculizumab, the highest number of deaths due to infection was seen in the study by Stephan et al. Based on their report, an increase in mortality due to infection in the group treated with Eculizumab was found compared to the conventional treatment group (21). Therefore, precaution and treatment of infection are equally urgent during the treatment of TA-TMA. GvHD is a risk factor that leads not only to TA-TMA but also to death during treatment. It has been shown that GvHD almost always precedes the diagnosis of TA-TMA, and there may be a mechanical link between TA-TMA, GvHD, and endothelial injury (33). Another study reported that the occurrence of TA-TMA was associated with risk factors such as aGvHD grade ≥2, steroid-refractory aGvHD, and CMV reactivation/end-organ disease, but not with conditioning regimen (RIC or MAC), TBI use or TBI dose, primary condition, donor type, age, or gender. More importantly, patients diagnosed with TA-TMA combined with aGvHD had significantly lower overall survival compared to patients with TA-TMA alone or GvHD (median 5.6 *vs.* 7.6 *vs.* 55.4 months; p < 0.0001) (34). The relationship between TA-TMA and GvHD is unclear. Future studies should provide information on the relationship between GvHD and Evidence of the TA-TMA link. TA-TMA itself is another major cause of death because of endothelial injury-related organ failure. It is necessary to explore how endothelial cells are damaged. One study from our center has reported that heme oxygenase-1 (HO-1) was significantly decreased in patients with TA-TMA and suppressed oxidative stress could attenuate complement deposition in TMA plasma-challenged HUVECs (35). The nuclear factor erythroid 2-related factor 2 (Nrf2), a transcription factor, initiates transcription of the HO-1 gene to protect cells from oxidative stress (36). Further experimental study about Nrf2 and endothelial injury is undertaken in our center.

Another area that needs to be discussed is how much Eculizumab needs to be given to achieve a hematological response and the duration of Eculizumab treatment for TA-TMA. For adult patients, the dose of induction therapy was 900 mg per week for four weeks. If the patient responds to induction therapy, the treatment is maintained at 1,200 mg administered every two weeks. For pediatric patients, the initial dose is based on body weight, and subsequent dose adjustments are based on maintaining total hemolytic complement activity (CH50) levels. Patients weighing less than 40 kg started at 600 mg and others at 900 mg. Induction therapy is also administered weekly for four weeks, and CH50 should be maintained at complement activator enzyme (CAE) levels of 0 to 3 (18, 37). The treatment then transitions to maintenance therapy, which adequately suppresses CH50 to a scale of three CAE (4) and then to maintenance therapy. Regarding the question of when Eculizumab can be safely discontinued, the study by Prajwal et al. proposed that ECU can be suspended after determining clinical symptoms and laboratory manifestations (38).

There are still some potential limitations to our study. First, there is a complete lack of randomized controlled trials and a limited study population size, and investigators have conducted limited studies on the efficacy of Eculizumab for TA-TMA. Second, although there is a great deal of heterogeneity among the included studies, the limited number of included studies prevents us from analyzing the sources of heterogeneity. Third, AEs are generalized in the article, so we do not have access to security data for AEs. Despite these limitations, our review is the first comprehensive meta-analysis of all eligible studies that analyzed the efficacy and safety of Eculizumab in patients with TA-TMA.

## Conclusion

This systematic review and meta-analysis suggest that Eculizumab improves SR and ORR in patients with TA-TMA. Furthermore, patients with TA-TMA diagnosed within the first 100 days after HSCT are more likely to achieve better outcomes with Eculizumab compared to patients with TA-TMA diagnosed more than 100 days after HSCT. In addition, Eculizumab is well-tolerated, but the prevention and treatment of infection still require attention. Further RCTs and extensive prospective cohort studies are needed to evaluate efficacy and safety, particularly for Eculizumab for TA-TMA.

## Data Availability Statement

The datasets presented in this study can be found in online repositories. The names of the repository/repositories and accession number(s) can be found in the article/**Supplementary Material**.

## Author Contributions

RZ, MZ, and JQ contributed to the study conception and design, and writing the manuscript. RZ, WM, and ZZ performed data collection and analysis. DW and YH commented on the research design, data analysis, writing the manuscript, and supervision of the study. All authors contributed to the article and approved the submitted version.

## Funding

This work was supported by the National Natural Science Foundation of China (81873432 and 81670132), grants from the Jiangsu Province of China (BE2016665, SBE2016740635 and ZDRCA2016047), The Natural Science Foundation of the Jiangsu Higher Education Institution of China (18KJA320006), and the Priority Academic Program Development of Jiangsu Higher Education Institutions (PAPD).

## Conflict of Interest

The authors declare that the research was conducted in the absence of any commercial or financial relationships that could be construed as a potential conflict of interest.
