# Supplementary material for: Efficacy and Safety of Eculizumab in the Treatment of Transplant-Associated Thrombotic Microangiopathy: A Systematic Review and Meta-Analysis
Source: Front Immunol. 2021 Jan 20;11:564647. doi: 10.3389/fimmu.2020.564647 (PMC7856300; doi:10.3389/fimmu.2020.564647)
Supplement: Supplementary file 1 [file Table_1.docx]

**Supplementary Table 1.** stratified analyses of overall response rate (ORR) of TA-TMA patients after Eculizumab treatment.

| Stratified analyses | No. of studies | Pooled ORR (95%CI) | Heterogeneity | Metaregression (p-value) |
| --- | --- | --- | --- | --- |
| All observational studies | 6 | 0.71 (0.58, 0.82) | I^2^=30%, p=0.21 |  |
| Setting |  |  |  | 0.4867 |
| Multicenter | 2 | 0.63(0.35,0.87) | I^2^=33%, p=0.22 |  |
| Single-centre | 4 | 0.74(0.57,0.88) | I^2^=42%, p=0.16 |  |
| Sample size |  |  |  | 0.9000 |
| <10 | 2 | 0.74(0.47,0.94) | I^2^=0%, p=0.65 |  |
| ≥10 | 4 | 0.70(0.52,0.86) | I^2^=56%, p=0.08 |  |
| Median age |  |  |  | 0.6907 |
| <18 | 3 | 0.67 (0.55, 0.78) | I^2^=0%, p=0.78 |  |
| ≥18 | 3 | 0.73 (0.44, 0.95) | I^2^=67%, p=0.05 |  |
| Primary disease |  |  |  | 0.6907 |
| hematological disease | 3 | 0.73 (0.44, 0.95) | I^2^=67%, p=0.05 |  |
| hematological disease/others | 3 | 0.67 (0.55, 0.78) | I^2^=0%, p=0.78 |  |
| Days between transplant to TA-TMA |  |  |  | 0.6276 |
| <100 | 4 | 0.67 (0.56, 0.77) | I^2^=0%, p=0.91 |  |
| ≥100 | 2 | 0.74 (0.27, 1.00) | I^2^=83%, p=0.02 |  |
| Eculizumab as first-line or second-line |  |  |  | 0.5013 |
| First-line | 2 | 0.65 (0.53, 0.77) | I^2^=0%, p=0.98 |  |
| First-line/second-line | 4 | 0.74 (0.53, 0.92) | I^2^=50%, p=0.11 |  |
| Therapy duration |  |  |  | 0.0558 |
| <60 | 2 | 0.84 (0.57, 1.00) | I^2^=48%, p=0.17 |  |
| ≥60 | 4 | 0.64 (0.54, 0.75) | I^2^=0%, p=0.67 |  |
| Number of Eculizumab doses |  |  |  | 0.3050 |
| <8 | 2 | 0.59 (0.37, 0.80) | I^2^=0%, p=0.37 |  |
| ≥8 | 4 | 0.75 (0.58, 0.89) | I^2^=43%, p=0.15 |  |

**Supplementary Figure 1.** Sensitivity analysis for overall response rate (ORR) of the TA-TMA patients after Eculizumab treatment.

**Supplementary Figure 2.** Sensitivity analysis for complete response rate (CRR) of the TA-TMA patients after Eculizumab treatment.

**Supplementary Table 2.** stratified analyses of survival rate (SR) of TA-TMA patients after Eculizumab treatment.

| Stratified analyses | No. of studies | Pooled SR (95%CI) | Heterogeneity | Metaregression (p-value) |
| --- | --- | --- | --- | --- |
| All observational studies | 6 | 0.52 (0.40, 0.65) | I^2^=24%, p=0.25 |  |
| Publication year |  |  |  | 0.3546 |
| <2018 | 3 | 0.47 (0.20, 0.74) | I^2^=60%, p=0.08 |  |
| ≥2018 | 3 | 0.57 (0.45, 0.68) | I^2^=0%, p=0.87 |  |
| Setting |  |  |  | 0.9201 |
| Multicenter | 2 | 0.55 (0.14,0.93) | I^2^=74%, p=0.05 |  |
| Single-centre | 4 | 0.53 (0.42,0.63) | I^2^=0%, p=0.42 |  |
| Sample size |  |  |  | 0.0975 |
| ≤10 | 3 | 0.68 (0.47,0.86) | I^2^=0%, p=0.74 |  |
| >10 | 3 | 0.45 (0.29,0.61) | I^2^=39%, p=0.20 |  |
| Median age |  |  |  | 0.0827 |
| <18 | 3 | 0.59 (0.47, 0.70) | I^2^=0%, p=0.43 |  |
| ≥18 | 3 | 0.40 (0.24, 0.57) | I^2^=0%, p=0.38 |  |
| Primary disease |  |  |  | 0.0827 |
| hematological disease | 3 | 0.40 (0.24, 0.57) | I^2^=0%, p=0.38 |  |
| hematological disease/others | 3 | 0.59 (0.47, 0.70) | I^2^=0%, p=0.43 |  |
| Days between transplant to TA-TMA |  |  |  | 0.0266 |
| <100 | 4 | 0.59 (0.48, 0.70) | I^2^=0%, p=0.64 |  |
| ≥100 | 2 | 0.33 (0.16, 0.53) | I^2^=0%, p=0.99 |  |
| Therapy duration |  |  |  | 0.0827 |
| ≤65 | 3 | 0.40 (0.24, 0.57) | I^2^=0%, p=0.38 |  |
| >65 | 3 | 0.59 (0.47, 0.70) | I^2^=0%, p=0.43 |  |
| Number of Eculizumab doses |  |  |  | 0.5498 |
| <8 | 2 | 0.46 (0.21, 0.71) | I^2^=29%, p=0.23 |  |
| ≥8 | 4 | 0.55 (0.39, 0.71) | I^2^=36%, p=0.101 |  |

**Supplementary Figure 3.** Sensitivity analysis for survival rate (SR) of the TA-TMA patients after Eculizumab treatment.

**Supplementary Table 3.** Newcastle-Ottawa scale scores.

| Study | Selection | Comparability | Outcome | Total |
| --- | --- | --- | --- | --- |
| Flore et al | 4 | 1**^b^** | 2**^c^** | 7 |
| Prajwal et al | 3**^a^** | 1**^b^** | 3 | 7 |
| Stephan et al | 4 | 2 | 3 | 9 |
| Joslyn et al | 3**^a^** | 2 | 3 | 8 |
| Michelle et al | 4 | 1**^b^** | 2**^c^** | 7 |
| Sonata et al | 4 | 1**^b^** | 3 | 8 |

Reasons for lost stars:

**a.** the non-exposed cohort drawn from a different source or no description of the derivation of the non-exposed cohort.

**b.** study not controlling other additional factors, such as age, primary disease, type of transplant.

**c.** follow-up not long enough for outcomes to occur.
